# The Ion Formation and Quantitative Response of Isoprene, Monoterpenes and Terpenoids in Ion Mobility Spectrometry with Atmospheric-Pressure Chemical Ionization as a Function of Temperature

**DOI:** 10.3390/s24247976

**Published:** 2024-12-13

**Authors:** Thomas Mayer, Ralf Petrich, Helko Borsdorf

**Affiliations:** 1 UFZ Helmholtz Centre for Environmental Research, Department Monitoring and Exploration Technologies, Permoserstraße 15, 04318 Leipzig, Germany; thomas.mayer@ufz.de; 2 IFU GmbH Private Institute for Analytics, An der Autobahn 7, 09669 Frankenberg/Sa., Germany; ralf.petrich@ifu-analytik.de

**Keywords:** ion mobility spectrometry, atmospheric-pressure chemical ionization, isoprene, monoterpenes, terpenoids

## Abstract

Ion mobility spectrometry is successfully used as a sensor technology for different applications. A feature of this method is that characteristic ion mobility spectra are obtained for each measurement rather than a sum signal. The spectra result from the different drift velocities of ions in a drift tube at atmospheric pressure. In this study, we investigated the ion formation processes and the quantitative response of isoprene, monoterpenes and monoterpenoids as a function of the temperature of the spectrometer using a tritium ionization source. These substances are important target analytes in atmospheric monitoring and in the analysis of essential oils in different matrices. A drift tube temperature above 120 °C permitted the most sensitive detection of isoprene and monoterpenes, while 80 °C was sufficient for the sensitive detection of most terpenoids. Dimer ions were formed for isoprene over the whole temperature range. The ionization processes of monoterpenes and terpenoids were strongly influenced by the temperature. At temperatures of 40 °C, adduct ions were formed in addition to MH^+^ ions for monoterpenes. Enhanced temperatures provided a single peak with the same drift time for all monoterpenes. Structural differences influenced the ion formation of terpenoids, and much more complex spectra were obtained. The nature of the product ions changed depending on the temperature.

## 1. Introduction

Ion mobility spectrometry (IMS) is a well-established sensor technique for fast online and onsite measurements of volatile and semi-volatile organic compounds in ambient air. Configuration as handheld or field-portable spectrometers operating at atmospheric pressure, measurements in real time and low detection limits down to the ppb_V_ or even the ppt_V_ range have broadened its range of applications [1,2]. While IMS was solely used as a military chemical-agent detector or drug test in the past [3,4], it is nowadays a well-established sensor technique for civilian and non-security applications, and it is routinely applied in environmental monitoring [5,6], food safety [7,8], process control [9], clinical diagnostics [10], pharmacy [11] and biomedical science [12]. The key advantage of ion mobility spectrometry as a sensor technology is that the ion mobility spectra are detected for each scan rather than sum signals only, as is the case for nearly all low-cost sensors (e.g., electrochemical or metal oxide sensors) [13].

Ion mobility measurements are based on the determination of the drift velocities (v_d_) of ion swarms derived from sample molecules. Since the measurement is made on ions, the formation of ions from neutral sample molecules is the first and the controlling event in the method. The ionization of the sample occurs in air at ambient pressure. The common method for ionization in conventional ion mobility analyzers is through chemical reactions between the sample and reactant ions arising from the emission of electrons from radioactive nickel (^63^Ni), tritium (^3^H) or a corona discharge (CD) into a supporting atmosphere. The formed ions are injected at a given time interval of a few microseconds via an electronic shutter into the drift region. Traditional drift tubes consist of circular metal rings that are separated by insulators. Drift rings are attached to a voltage divider that provides differences in potential between the rings and establishes the electric field. Typical voltage gradients are in the range between 200 and 400 V cm^−1^ with 5 to 20 cm long drift tubes. The ion packet in the drift region moves as a swarm toward a detector (typically, a Faraday plate) down the voltage gradient and through a gas atmosphere, air or nitrogen. The ion swarm has characteristic drift velocities, providing a basis for the separation of ions through differences in mass and structure. The resulting ion mobility spectrum shows the arrival time distribution of the formed ions at the detector. The drift velocity (v_d_) of separate ions is proportional to the field strength (E) and, when normalized to the unit field strength, is termed the mobility coefficient (K = v_d_ E^−1^ in units of cm^2^ Vs^−1^). The mobility coefficient normalized to 273 K and 760 Torr (mm Hg) is called the reduced mobility coefficient (K_0_) [14].

One important class of target analytes is terpenes. Monoterpenes consist of two isoprene units with a molecular formula of C_10_H_16_. They exhibit a wide range of isomeric structures. Terpenoids are derived from terpenes through oxygenation. Isoprene and monoterpenes are biosynthetically formed within plants via two synthesis pathways: the mevalonic acid pathway, which takes place in the microsomes and cytoplasm, and the methylerythritol phosphate pathway, which takes place in the chloroplasts [15]. Vegetation emits significant amounts of terpenes into the atmosphere, influencing ecological interactions and atmospheric chemistry. Furthermore, terpenes are the major components of essential oils extracted from plants. Essential oils are widely used in the pharmaceutical, sanitary, cosmetic, agricultural and food industries [16,17,18].

In these contexts, ion mobility spectrometry has been successfully used for the detection of emission patterns from plants [19,20], in food flavor analysis [8] and for the characterization of essential oils [21] and natural products [22]. Terpenes are among the main components in all these matrices. Due to the structural complexity of terpenes, IMS is often used in combination with gas-chromatographic pre-separation. Various chemometric methods have been developed for the faster evaluation of complex chromatograms [21,23]. When using IMS with a tritium source without chromatographic pre-separation, previous studies have shown that the selectivity of the measurement can be significantly improved by dopants. The addition of NO as a dopant led to the formation of additional product ions and increased the selectivity of the ion mobility measurement [24].

**Figure 1:**
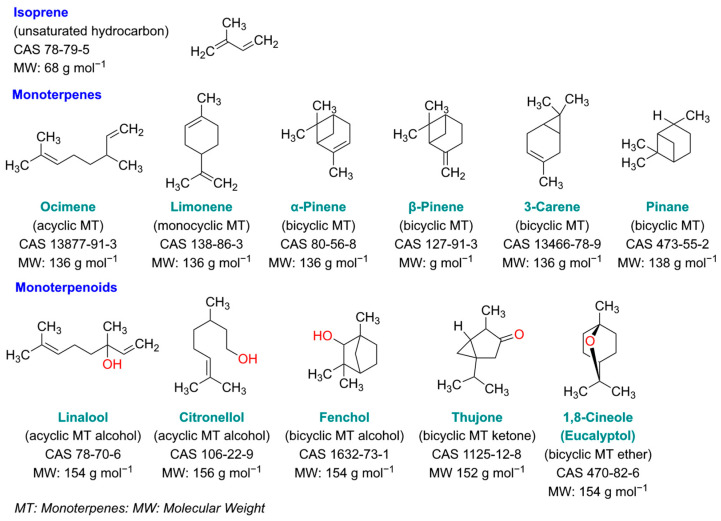
Structures of investigated substances.

Whether the ion mobility spectrometer is used as a separate sensor or as a detector for gas chromatography, it is necessary to understand the resulting ion mobility spectra and to find the conditions for the most sensitive detection of these compounds. Humidity and temperature are key parameters that influence the qualitative and quantitative response in IMS [25,26]. Since the influence of humidity is minimized when coupling IMS with gas chromatography, we focused on investigating the influence of temperature in this study. The scientific question for these investigations was to assess how structural differences influence ion formation and in which way different temperatures have influence on the positions of product ion peaks and the quantitative response. For this purpose, isoprene and different monoterpenes and monoterpenoids were investigated. The monoterpenes were selected so that acyclic (ocimene), monocyclic (limonene) and saturated (pinane) and unsaturated bicyclic monoterpenes were included. The unsaturated bicyclic monoterpenes differ in the position of the double bond, inside (α-pinene, 3-carene) or outside (β-pinene) the carbon ring. Terpenoids have additional functional groups, containing oxygen. We investigated acyclic monoterpene alcohols (linalool, citronellol), a bicyclic monoterpene alcohol (fenchol), a bicyclic monoterpene ketone (thujone) and a bicyclic monoterpene ether (1,8-cineole). The structures of these compounds are summarized in Figure 1. The investigations presented here were carried out with a tritium ion source, as radioactive atmospheric-pressure chemical ionization using a β-emitter is still the most widely used ion source in IMS.

Detailed investigations on the ion formation of terpenes in ion mobility spectrometry have been carried out mainly using photoionization and CD ionization [27,28]. Photoionization provides ion mobility spectra containing one major peak for saturated compounds, while two peaks are observed for unsaturated compounds, which can be assigned to product ions related to monomer and dimer ions. Differences in the relative abundance of product ions were found depending on structural features. Although IMS using CD ionization permits the most sensitive detection, the spectra are complex and differ from those obtained using photoionization due to the formation of additional cluster ions and fragment ions. 

Temperature effects in IMS have been investigated for different classes of compounds [26,29]. However, these studies have not been carried out for terpenes. Furthermore, previous investigations of the temperature effect have been limited to studies of the relationship for drift time vs. temperature without consideration of the quantitative effects. 

## 2. Materials and Methods

The substances used in this study (isoprene, unsaturated monocyclic terpenes, unsaturated and saturated bicyclic terpenes, monoterpenoids) had a purity of about 99% and were obtained as a racemic mixture from Sigma Aldrich, St. Louis, MO, USA, or Glentham Life Sciences Ltd., Corsham, UK.

Since no permeation tubes were commercially available for the terpenes to be examined, self-made permeation vials were used. Briefly, 2 mL screw vials (glass) were filled with 500 µL of substance. The original septa were replaced by the desired membrane materials. Depending on the permeation rates of the different compounds, we used polyethylene (PE) with a thickness 300 µm or polydimethylsiloxane (PDMS) with a thickness 250 µm. Before weighing, the vials were pre-permeated for 24 hours for equilibration. 

The samples were introduced via a self-developed reference gas generator, which has been described in a previous publication [30]. The permeation vessels were placed in a permeation cell. A constant gas flow passed the permeation cell, which was also temperature-controlled. The temperatures were adjustable between <10 °C and 100 °C using a Peltier element (QC-PC-CO-CH1, Quick-Ohm Küpper & Co. GmbH, Wupperthal, Germany). An aliquot was taken from this gas stream, transferred to a mixing chamber and further diluted by an additional gas supply. This resulting gas flow was divided, and a certain aliquot of the diluted gas stream was transferred to the ion mobility spectrometer. The flow rate of this aliquot was variable and could be adjusted to the requirement of the ion mobility spectrometer used. All the gas flows were adjusted via mass flow controllers. All parts of the reference gas generator that came into contact with the sample gas were made from high-alloy stainless steel (material 1.4401) and were thermostatically controlled at 70 °C. A detailed technical description is given in Ref. [30]. Nitrogen of 5.0 quality (with maximum concentrations of <3 ppm oxygen and <3 ppm water according to the manufacturer’s specification) was used as the transport gas and the diluent gas. 

The ion mobility measurements were performed with a commercially available spectrometer from STEP Sensortechnik und Elektronik Pockau GmbH (Pockau, Germany). The device is equipped with a tritium ionization source (50 MBq) and works with a unidirectional flow system. Nitrogen of 5.0 quality was used as the drift gas with a flow rate of approximately 400 mL min^−1^. The ions formed were gated to the drift tube via a 60 µs impulse, which was applied to the ion source. An additional grid with the same potential as the first drift ring was located approximately 1 mm apart from the ion source. The drift tube consisted of a coated ceramic tube with a length of 5.6 cm. An electric field of 300 V cm^−1^ was applied. The spectrometer could operate in a temperature range between 40 and 150 °C. The data acquisition and the analysis of spectra were performed using IMS Control Basic software V 2.0 (STEP Sensortechnik und Elektronik Pockau GmbH, Pockau, Germany). All the temperature-dependence measurements were carried out with this spectrometer.

Additional measurements and the analysis of mixtures were performed with a Smellmaster^®^ 2 ion mobility spectrometer (IFU GmbH Privates Institut für Analytik, Frankenberg, Germany) equipped with a tritium ionization source. The following operational conditions were applied for the measurements: ionization source: tritium (50 MBq); shutter opening time: 100 µs; length of drift tube: 5.6 cm; drift voltage: 2470 V; polarity of ions measured: positive; drift gas: scrubbed air with 340 mL min^−1^ guided within a closed circuit; drift tube temperature 80 °C; pressure: ambient (950–1050 hPa). 

## 3. Results and Discussion

### 3.1. Peak Positions Depending on Temperature

#### 3.1.1. Isoprene and Monoterpenes

**Figure 2:**
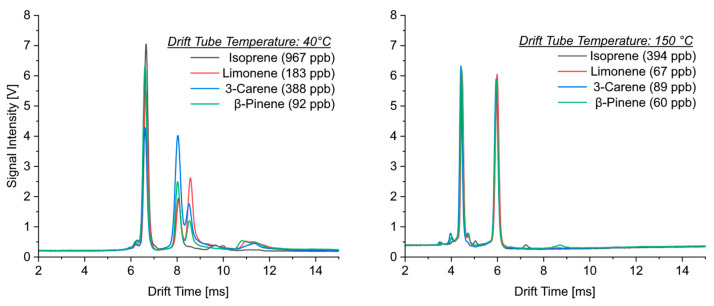
Ion mobility spectra of isoprene, limonene, 3-carene and β-pinene taken at drift tube temperatures of 40 °C (**left**) and 150 °C (**right**).

Figure 2 shows the ion mobility spectra of isoprene and selected monoterpenes obtained at different temperatures (40 °C and 150 °C). Some fundamental differences can be derived from the comparison of these spectra. The most important parameter influencing the drift time is the molecular weight. However, although the molecular weight of isoprene (68 g mol^−1^) is half of that of monoterpenes (136 g mol^−1^), the drift times were the same. In addition to the reactant ion peak (the left peak in all the ion mobility spectra), all four substances showed one product ion peak with nearly identical drift times (between 5.96 and 6.03 ms) at 150 °C. It was not possible to differentiate between isoprene and monoterpenes or between the monoterpenes themselves at this temperature. Even at 40 °C, only one product ion peak formed for isoprene, while two product ion peaks were observed for monoterpenes. The drift times of the product ion peaks with higher mobility were also identical for both isoprene and monoterpenes (between 8.02 and 8.09 ms). Since ion mobility spectrometry is usually able to clearly separate such mass differences, it can be assumed that isoprene formed dimer ions over the whole temperature range. The dimerization of isoprene was also observed in measurements using gas chromatography/mass spectrometry coupling, although it is not clear where this reaction took place, either in the inlet of the gas chromatograph [31] or at the ion source of the mass spectrometer [32].

A second product ion peak with a lower mobility was observed in the ion mobility spectra of monoterpenes at a temperature of 40°. This additional peak had its maximum intensity at drift times between 8.52 and 8.59 ms. The difference in drift times between both product ion peaks was approximately 0.5 ms. Except for ocimene, both peaks were well resolved and separated. For nearly all the investigated monoterpenes, the intensity of this second product ion peak was slightly lower than that for the more mobile ion. 

A deviating behavior was observed for limonene, for which the additional adduct ion peak had higher intensities at low temperatures. The comparison of the drift times of the isoprene dimer M_2_H^+^ with an ionic mass of 137 with those of the protonated ions of monoterpenes MH^+^ (also with an ionic mass of 137) indicates that the more mobile ions of monoterpenes resulted from protonation, while the additional ions could be attributed to the formation of adduct ions. 

**Figure 3:**
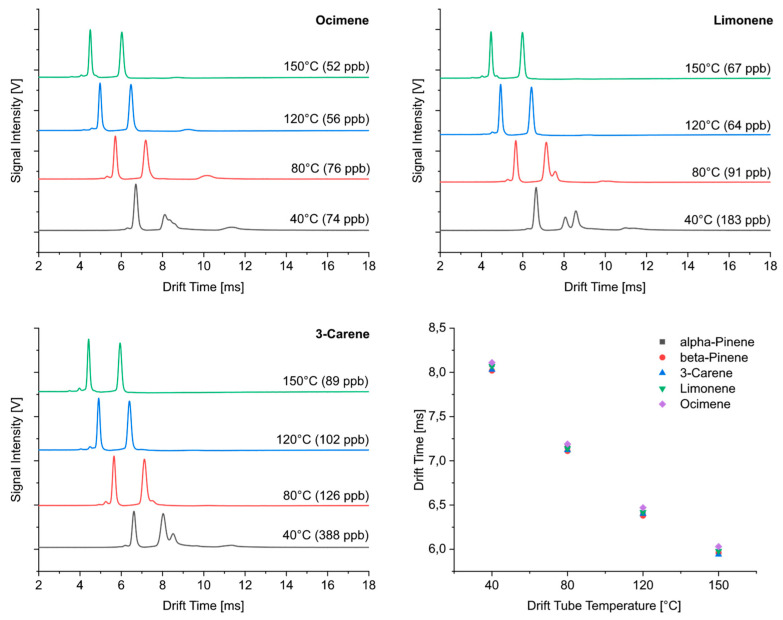
Ion mobility spectra of selected monoterpenes taken at different drift tube temperatures and dependence of drift times on temperature.

Figure 3 shows the ion mobility spectra obtained at different temperatures more in detail for selected monoterpenes. As expected, the drift times generally decreased with increasing temperatures. An increase in temperature from 40 °C to 150 °C caused a shift in the drift times of the MH^+^ product ion peak from approximately 8 ms to 6 ms. Furthermore, the temperature dependence for this peak was identical for all the monoterpenes investigated here. The additional adduct ion peak was only observable at low temperatures; its intensity decreased with increasing temperature and could not be detected at temperatures above 80 °C.

The preferred formation of MH^+^ ions at higher temperatures and the occurrence of additional adduct ions detected at lower temperatures obviously resulted from the physicochemical properties of the reactant ions and target analytes. Data on the proton affinities of monoterpenes are only available to a very limited extent. The data available in the literature are summarized in Table 1.

Highlighted in red are the effective proton affinities of the hydrated forms of the reactant ions. Since our carrier gas and the drift gas had a constant humidity of 3 ppm, the distribution of the clusters mainly depended on the temperature. The degree of clustering for H^+^(H_2_O)_x_ reactant ions depending on the temperature can be calculated using the known enthalpies (ΔH^0^) and entropies (ΔS^0^) of hydration [36,37]. As known, a proton transfer from the reactant ion to the analyte molecule only takes place if the proton affinity of the analyte is higher than that of the reactant ion. Considering the data in Table 1, proton transfer from the hydrated reactant ions to the monoterpenes can only occur if (H_3_O)^+^ or (H_3_O)^+^(H_2_O) reactant ions are present in the ion source. At temperatures of 40 °C, mainly (H_3_O)^+^(H_2_O)_2_ and (H_3_O)^+^(H_2_O)_3_ reactant ions are formed, which do not permit a direct proton transfer to the monoterpene molecules. The formation of (H_3_O)^+^(H_2_O) reactant ions starts at 70 °C, and their relative abundance increases with higher temperatures while fewer (H_3_O)^+^(H_2_O)_2_ reactant ions are formed with increasing temperature. The increasing supply of (H_3_O)^+^(H_2_O) reactant ions with elevated temperatures and the possibility of direct proton transfer explains the formation of one single peak at temperatures above 80 °C, which can be assigned to MH^+^ product ions. The additional adduct ions detected at lower temperatures can obviously be assigned to water clusters M(H_2_O)H^+^ due to their drift time difference compared with MH^+^ ions and their occurrence only at lower temperatures. If these assumptions are correct, the measurements at elevated temperatures should also be much more sensitive than those at lower temperatures (see Section 3.2). The higher intensity of M(H_2_O)H^+^ product ions at low temperature in the case of limonene obviously resulted from the occurrence of two double bonds in comparison with the other monoterpenes. These localized charges apparently led to an improved attachment of water. 

**Figure 4:**
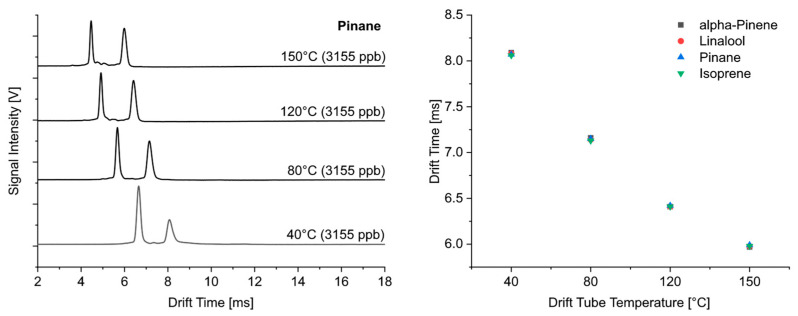
Ion mobility spectra of pinane taken at different drift tube temperatures and dependence of drift times of isoprene, pinane, α-pinene and linalool on temperature.

We would like to show with Figure 4 that there were no differences in the temperature dependence of the drift times for isoprene, saturated (pinane) and unsaturated (α-pinene) monoterpenes and linalool as an acyclic monoterpene alcohol. The main peak of all these compounds appeared at the same drift time in the ion mobility spectrum for each temperature, and, consequently, the temperature dependence for these substances was identical.

#### 3.1.2. Terpenoids

**Figure 5:**
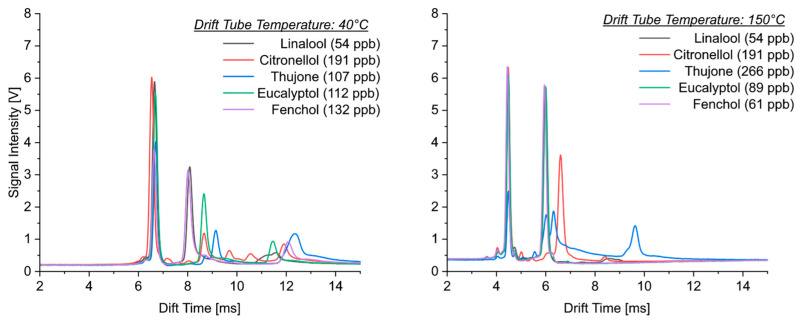
Ion mobility spectra of linalool, citronellol, thujone, eucalyptol and fenchol taken at drift tube temperatures of 40 °C (**left**) and 150 °C (**right**).

As shown in Figure 4, the product ions of linalool, an acyclic monoterpene alcohol with a molecular weight of 154 g mol^−1^, appeared at the same drift time and with the same temperature dependence in the ion mobility spectra as those for isoprene (68 g mol^−1^) and monoterpenes (136 g mol^−1^). It can, therefore, be assumed that the -OH group was eliminated during the ionization reaction in the case of linalool. However, the comparison of the ion mobility spectra (Figure 5) shows clear differences between the substances studied, both at low and high temperatures.

Linalool and fenchol formed one product ion at 40 °C, which appeared at the same drift time. All the other investigated monoterpenoids formed more product ions, providing a series of peaks. The most intense product ion peaks of citronellol and eucalyptol also had the same drift time but were different from those of linalool and fenchol. At 150 °C, these four substances showed one intense product ion peak. The drift times of this peak were comparable for linalool, fenchol and eucalyptol (approximately 6 ms), while the product ions of citronellol were detected at 6.6 ms. Thujone showed a deviating behavior. At 40 °C, a small product ion peak was detectable at the same drift time as that observed for citronellol and eucalyptol. The most intense peak appeared at 9.1 ms. No other terpenoid showed peaks at this position. A third peak could be observed > 12 ms. At 150 °C, the most mobile ion had the same drift time as that observed for linalool, fenchol and eucalyptol. A second peak was detected at 6.3 ms, where no other terpenoid provided product ion peaks. The third peak shifted to 9.3 ms. 

**Figure 6:**
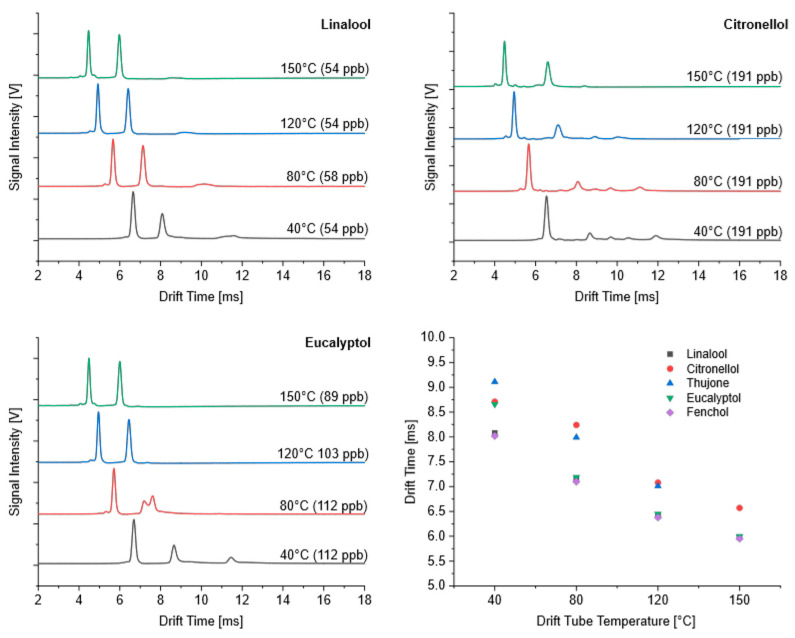
Ion mobility spectra of selected monoterpenoids taken at different drift tube temperatures and dependence of drift times on temperature.

**Figure 7:**
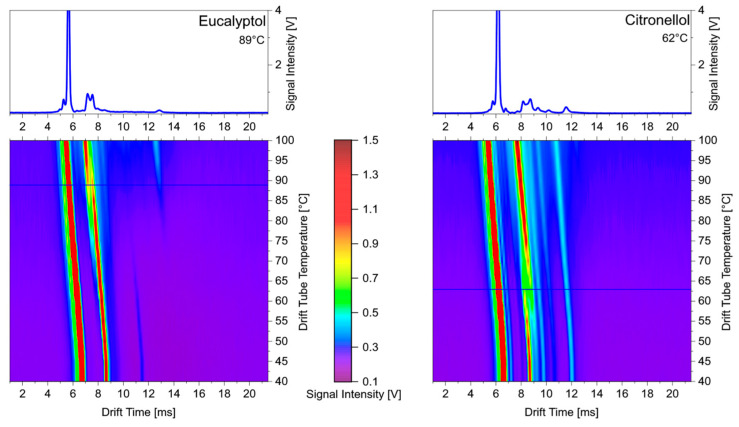
Ion mobility spectra of eucalyptol and citronellol depending on temperature.

Fenchol and linalool mainly formed product ions with the same drift time and temperature dependence. As described above, the detected drift times at different temperatures were identical to those of monoterpenes. Therefore, the cleavage of the OH group can be assumed for both substances over the whole temperature range. Looking at the spectra of eucalyptol in Figure 6, the peak splitting of the more mobile ion at 80 °C is noticeable. At 40 °C, eucalyptol yielded product ions, whose drift time was similar to that of citronellol but different from those of fenchol and linalool. Surprisingly, the drift times of the more mobile ion at 80 °C and the product ion peaks above 80 °C corresponded to those of linalool and fenchol, respectively. From these observations, it can be assumed that MH^+^ ions formed at lower temperatures and that the cleavage of the OH group was only initiated at higher temperatures. The formation of MH^+^ product ions can be also supposed for citronellol at 40 °C due to the same drift times in comparison with eucalyptol. However, the temperature dependence of citronellol showed a different trend from the product ion peaks that were detectable at longer drift times. This contradictory behavior becomes clearer when looking in detail at the heating curve of the IMS with the corresponding ion mobility spectra. These are shown in Figure 7.

The course of the intensities of the product ions over temperature clearly showed a sharp transition from one product ion to another for both substances within a limited temperature range. If MH^+^ ions are formed for both substances at low temperatures, there is a certain temperature threshold for eucalyptol at which fragment ions are preferentially formed by the cleavage of the OH group. In the case of citronellol, no fragment ions are formed above this temperature threshold, but adduct ions are formed. This is clearly indicated by the shifting of the peaks to longer drift times. Unfortunately, it is not possible to explain this different behavior by differences in the structural features or the physicochemical data.

The most complex ion mobility spectrum at 150 °C was observed for thujone with three product ions. According to our observations described above, the peak of the most mobile ion obviously resulted from the formation of a fragment ions; the second peak could be attributed to MH^+^ ions, while the peak with longest drift time could be a dimer. 

In addition to the described peaks, other product ions in the expected drift time range of the dimers were detected at low temperatures for all the investigated monoterpenoids. Due to the considerable variation in the drift times for the individual substances, the structures causing this peak could not be clearly assigned.

### 3.2. Quantitative Response at Different Temperatures

**Figure 8:**
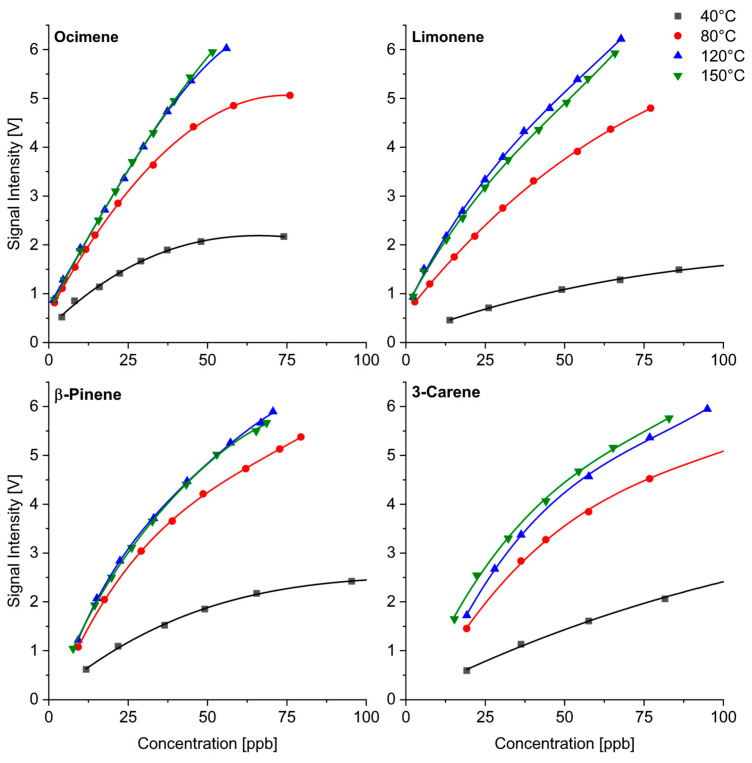
Calibration of selected monoterpenes as a function of temperature.

As described above, the proton transfer from (H_3_O)^+^(H_2_O) reactant ions to the terpenes is a feasible ionization reaction taking into account the data published in the literature for the proton affinities [35]. These ions are formed in higher quantities at temperatures above 70 °C. If proton transfer is the preferred ionization pathway, significantly better sensitivities should be expected at elevated temperatures. As shown in Figure 8 for unsaturated monoterpenes, we observed an increase in sensitivity with increasing temperatures up to 120 °C. The calibration functions at 120 °C and 150 °C had a similar slope for all the substances investigated. The evaluation of the spectra measured at 40 °C requires the consideration of all peaks due to the additional formation of adduct ions. Even if the sum of the intensities of all product ion peaks was considered (Figure 8), the measurements performed at 40 °C were less sensitive. While slight differences in the sensitivity between the different monoterpenes could be observed at lower temperatures, the measurements at 120 °C and 150 °C provided similar concentration dependencies. The detection limits of the unsaturated monoterpenes were in the low ppb range. Unfortunately, there are no real comparisons of the quantitative response with other ion sources in IMS, since previous studies focused mainly on the investigations of ionization reactions with photoionization and CD ionization. 

**Figure 9:**
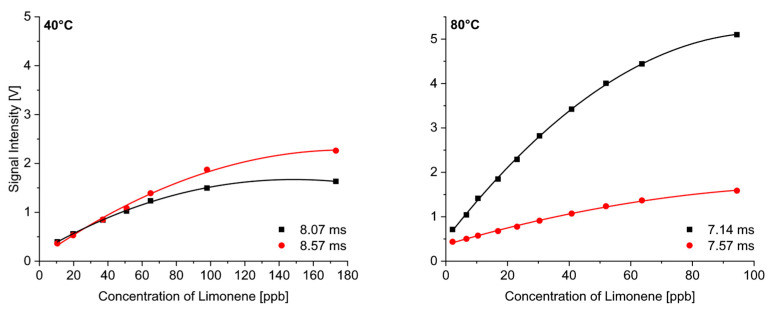
Quantitative response of the two product ion peaks of limonene at 40 °C and 80 °C.

The monoterpenes formed MH^+^ and additional M(H_2_O)H^+^ product ions, which were observable at low temperatures only. The adduct ions were formed in higher abundance for limonene than for the other monoterpenes. Therefore, we considered the concentration dependence of these two product ions for limonene separately, and the results are shown in Figure 9 for the measurements at 40 °C and 80 °C. The more mobile ion (black line) was MH^+^, while the heavier adduct ion (red line) with a supposed structure of M(H_2_O)H^+^ had lower mobility.

Limonene at 40 °C formed slightly more adduct ions, while an increasing temperature led to the preferred formation of MH^+^ product ions. The conclusion of this consideration is that the quantitative evaluation must include all the product ions, which makes the procedure more complex. Higher temperatures led to better sensitivities, and, above 80°, only one peak needed to be considered.

**Figure 10:**
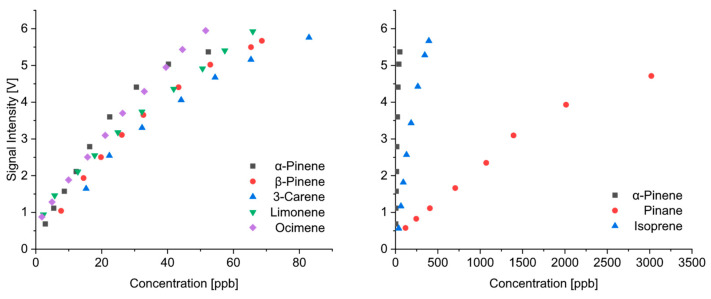
Comparison of quantitative response of unsaturated monoterpenes at 150 °C (**left**) and to a saturated monoterpene (pinane) and isoprene (**right**).

In Figure 10, we summarize the calibrations of all the unsaturated monoterpenes investigated here for a temperature of 150 °C (left). This comparison shows that all the unsaturated monoterpenes were detectable with similar sensitivities. While, so far, only the quantitative response of the unsaturated monoterpenes has been discussed, the right part of Figure 10 shows the comparison of the calibration of α-pinene with those of isoprene and pinane. The detection of pinane by IMS requires extremely high concentrations. Although no data for proton affinity are available in the literature, it is usual that saturated hydrocarbons have a much lower proton affinity than unsaturated substances. Therefore, ionization reactions are less effective when using β-emitters as ion sources. For this reason, saturated hydrocarbons are generally difficult to detect with this kind of an ion source. Isoprene is also less detectable than monoterpenes. This can be attributed to the lower proton affinity of isoprene in comparison with monoterpenes (see Table 1). 

**Figure 11:**
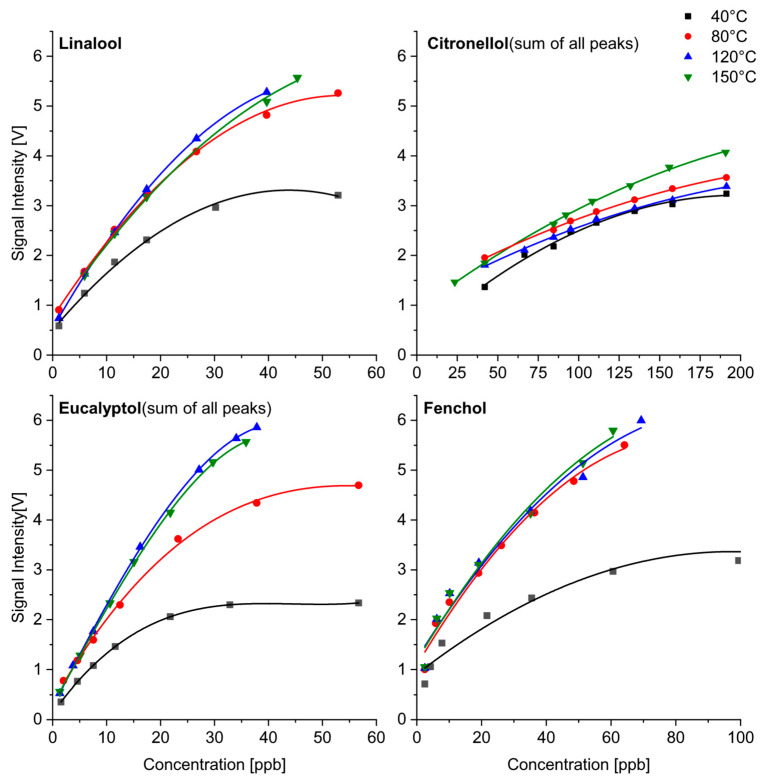
Calibration of selected monoterpenoids as a function of temperature.

Figure 11 shows the quantitative response of substances with one single peak over the whole temperature range (linalool, fenchol), two product ion peaks (eucalyptol) and a series of product ion peaks (citronellol). Fenchol and linalool showed lower peak intensities, depending on the concentration, at 40 °C than at the other temperatures. In contrast to monoterpenes with the most sensitive measurements above 120 °C, the maximum sensitivity was already achieved at 80 °C for all the monoterpene alcohols (linalool, citronellol and fenchol). The calibration of eucalyptol (monoterpene ether) depended on the temperature in a similar way as observed for monoterpenes. The calibrations at 120 °C and 150 °C were comparable, while lower intensities were found at 80 °C. However, it should be noted that two product ion peaks were analyzed for eucalyptol up to 80 °C, while only one product ion was present at temperatures above 120 °C. For Figure 11, the intensities of both peaks were added, which is subject to a large error due to the different responses of product ions with different structures. The spectra of citronellol (shown in Figure 6) and thujone were difficult to analyze due to the occurrence of different product ions over the whole temperature range. For the above-mentioned reason, the evaluation could have a considerable error. Both substances require high concentrations for their detection with IMS, and the detection limits are much higher than those for linalool, eucalyptol and fenchol. The calibration curves showed a limited sensitivity. Furthermore, no significant differences were observed for the calibrations at different temperatures.

### 3.3. Possible Errors and Repeatability of Measurements

All the temperature effects described above were investigated under defined experimental conditions. We used nitrogen with a low humidity and oxygen content as the carrier and drift gas. We chose this approach in order to minimize possible influencing factors. If we had used air as the drift gas, for example, oxygen from the air could have caused structural disruptions due to a possible oxidation reaction. Therefore, our results are specific to our experimental conditions. It must be verified whether these temperature dependencies can be reproduced with other drift gases.

Another possible source of error results from a general practical limitation of the IMS: uncertainties in the temperature control. The temperature sensors are positioned on the outside of the drift tube on most devices. Depending on the materials used, their thicknesses and the diameter of drift tube, the temperature inside the tube can vary and is device specific. For our analyses, we could only use the drift tube temperature displayed on the spectrometer as a basis. The temperatures measured on the spectrometer varied only very slightly for measurements on different days. At least 30 measurements per temperature were carried out for each substance. For α-pinene, for example, the measurements at 40 °C showed deviations of ±0.45 K (1.11%); at 80 °C, of ±0.49 K (0.61%); at 120 °C, of ±0.43 K (0.35%) and, at 150 °C, of ±0.48 K (0.31%) over all the measurements. These values are in the same range as that obtained for the other substances. Therefore, the deviations in the detected drift times were also relatively insignificant. Table 2 shows the detected drift times for isoprene, pinene and linalool and the deviations over all the measurements as an example. 

All the standard deviations of the drift time were below 1.5%; most of them were below 1% and permitted a reproducible determination with our spectrometer.

Quantitative measurements are more challenging than reproducibility. Possible sources of error may arise on the one hand from the generation of the reference gas and on the other hand from the measurement itself. To determine the permeation rates, it is necessary to weigh the permeation vials before and after dosing. The difference in weight is usually in the range of a few milligrams. Weighing is, therefore, the most significant source of error during the generation of the reference gas. When the permeation rates generated in the reference gas generator were examined on three different days with a new sample weight each time, larger deviations were observed. For example, an average permeation rate of 103.76 ± 3.6 µg h^−1^ (3.5%) was obtained for isoprene, 17.98 ± 2.6 µg h^−1^ (18%) for α-pinene and 24.33 ± 0.6 µg h^−1^ (2.4%) for linalool. It can be seen that the generation of the reference gas was associated with greater uncertainties.

**Figure 12:**
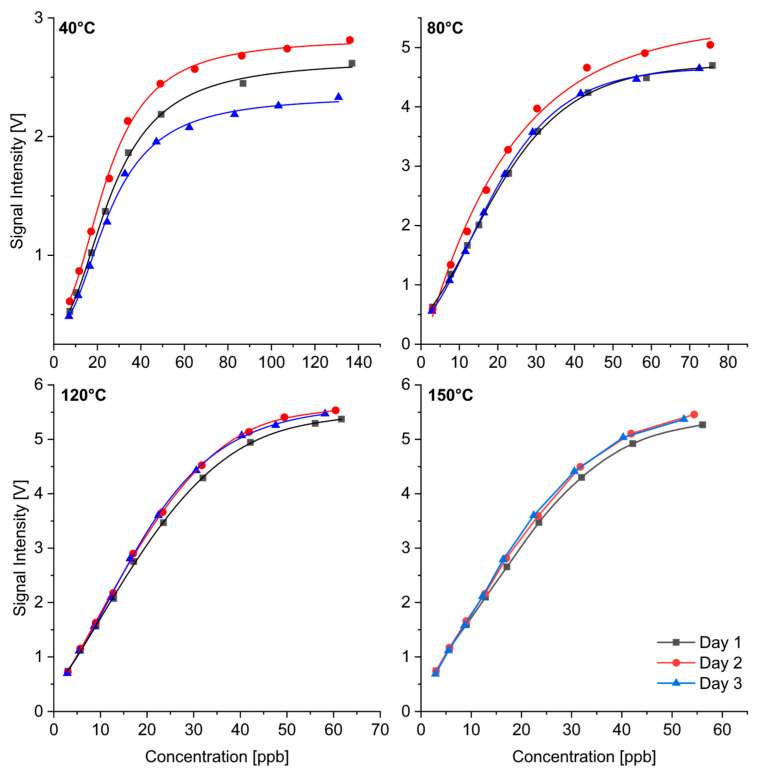
Repeatability of quantitative measurements of α-pinene on three days with new sample weight.

Figure 12 shows the repeatability of quantitative measurements of α-pinene on three days. It was difficult to determine exact standard deviations for a specific concentration, as the permeation method used provided slightly different concentrations if the measurements were carried out on different days with a new sample weight. Figure 12 shows that the repeatability improved with increasing temperature. Two reasons could explain this effect: The temperature stability of the spectrometer was better at higher temperatures; the deviations in the displayed temperature decreased with increasing temperature. The varying permeation rates also had an influence on the quantitative measurements. This becomes clear in the example of α-pinene with a standard deviation of 18% of the permeation rate.

It is generally difficult to generate concentrations of substances in the gas phase in the lower ppb range. However, the measurements on different days show that the differences in the drift times and in the quantitative response between the different substances were reproducible and repeatable.

### 3.4. Quantitative Response of Mixtures

Although it is known that the quantification of more than one substance in IMS is difficult due to possible competing reactions in the ion source, we investigated some binary mixtures of monoterpenes because their ionization reactions are similar. The question was whether the signal intensities behaved additively or whether a substance was preferentially ionized.

**Figure 13:**
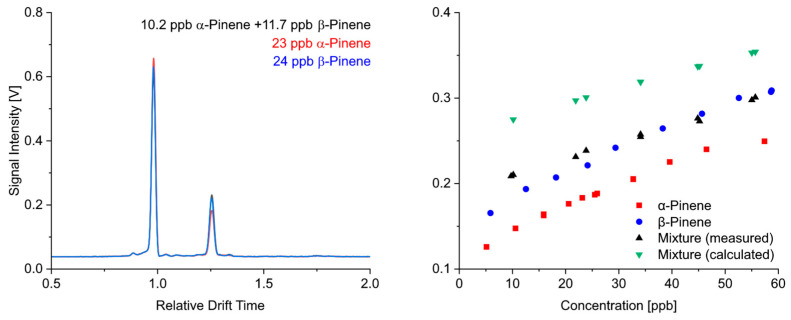
Ion mobility spectra and calibration of a mixture of α-pinene and β-pinene.

We chose α-pinene and β-pinene for this investigation due to their similarity in structure and physicochemical properties. Furthermore, the calibration curves of both the substances were comparable, and the mixtures could be adjusted so that comparable concentrations of both substances were injected. The left part of Figure 13 shows the ion mobility spectra of α-pinene, β-pinene and a mixture of both substances with comparable concentrations of each substance. It is clearly visible that the signal intensity of the mixture did not correspond to the sum of the intensities of the individual components. The calibration curves shown in the right part of Figure 13 confirm this. The signal intensities detected for the mixture corresponded very well with the signal intensities obtained for pure β-pinene. The expected signal intensities (sum of the intensities of the individual substances) were much higher (green dots). Obviously, the ions were preferentially formed from β-pinene. It seems that the difference in proton affinities of 10 kJ mol^−1^ was sufficient and that the substance with the higher proton affinity was preferentially formed, in our case β-pinene. No additive behavior regarding the quantitative response can, therefore, be expected even with structurally similar substances. 

## 4. Conclusions

The possibility of applying ion mobility spectrometry as a sensor technique for the quantitative determination of isoprene, monoterpenes and monoterpenoids depending on the temperature of the spectrometer using a tritium ionization source was investigated. Isoprene formed dimer ions over the whole temperature range between 40 °C and 150 °C. The sensitivity increased with increasing temperatures. The investigated unsaturated monoterpenes showed a comparable behavior in IMS, independent of the structural features. Low temperatures led to the formation of spectra consisting of protonated molecular ions and adduct ions. Enhanced temperatures permitted more sensitive detection, and single peaks were detected. Saturated monoterpenes were only insensitively detectable in comparison with unsaturated monoterpenes. The structural features of terpenoids had a more significant influence on the ion formation processes. Although linalool and citronellol are acyclic monoterpenes alcohols (citronellol has a terminal OH group in contrast to linalool), their ion mobility spectra were completely different. Thujone (bicyclic monoterpene ketone) and eucalyptol (bicyclic monoterpene ether) also provided spectra with different adduct ions, which formed in different intensities. Only fenchol and linalool (acyclic and bicyclic monoterpene alcohol) provided comparable spectra consisting of a single peak. At temperatures above 120 °C, only one single peak was detectable for all the investigated monoterpenoids (with the exception of thujone), and the measurements were more sensitive. The results can be summarized as follows: Low temperatures allowed substances to be distinguished to a certain extent by their different adducts. Higher temperatures provided spectra with a single peak, but it was not possible to distinguish between isoprene and the different monoterpenes. Citronellol and thujone could be distinguished from the other terpenoids at 150 °C. The measurement at higher temperatures was generally much more sensitive.

Independent of the use of ion mobility spectrometry as sensor technology for field applications, powerful methods for laboratory analysis are available. The traditional approach for determining terpenes in gaseous samples is the pre-concentration of analytes on sorbent tubes and a subsequent analysis in the lab using thermodesorption–gas chromatography—mass spectrometry (TD-GC-MS). Due to the possible variability of sampling time and sample gas streams, the detectable concentration ranges can be adjusted according to the requirements. Therefore, this approach is much more sensitive than field-deployable sensors with a direct analysis without sample preparation. Furthermore, mass spectrometry permits differentiation between most of the analytes due to different fragmentation patterns. On the other hand, these sensitive methods do not allow real-time monitoring and are much more expensive. 

## Figures and Tables

**Table 1:** Effective proton affinities of the hydrated forms of reactant ions (red) and target analytes.

Substance	Proton Affinity [kJ mol^−1^]	Reference
(H_3_O)^+^	691	[33]
(H_3_O)^+^(H_2_O)_1_	827	[33]
(H_3_O)^+^(H_2_O)_2_	911	[33]
(H_3_O)^+^(H_2_O)_3_	984	[33]
Isoprene	826.4	[34]
Ocimene	881.6	[35]
α-Pinene	863.2	[35]
β-Pinene	873.2	[35]
3-Carene	859.4	[35]
Limonene	841.8	[35]

**Table 2:** Averaged drift times and standard deviations of at least 30 measurements per temperature.

Substance	40 °C	80 °C	120 °C	180 °C
Isoprene	8.09 ± 0.008	7.16 ± 0.010	6.42 ± 0.011	5.98 ± 0.009
α-Pinene	8.06 ± 0.093	7.14 ± 0.101	6.40 ± 0.090	5.95 ± 0.086
β-Pinene	7.96 ± 0.050	7.07 ± 0.044	6.34 ± 0.047	5.12 ± 0.045
Linalool	8.11 ± 0.017	7.16 ± 0.015	6.42 ± 0.010	5.99 ± 0.025

## Data Availability

The analytical results and data are available from the corresponding author upon request.
